# Coordination Chemistry of Ru(II) Complexes of an Asymmetric Bipyridine Analogue: Synergistic Effects of Supporting Ligand and Coordination Geometry on Reactivities

**DOI:** 10.3390/molecules25010027

**Published:** 2019-12-19

**Authors:** Komi Akatsuka, Ryosuke Abe, Tsugiko Takase, Dai Oyama

**Affiliations:** Cluster of Science and Engineering, Fukushima University, 1 Kanayagawa, Fukushima 960-1296, Japan; s1870001@ipc.fukushima-u.ac.jp (K.A.); ttakase@sss.fukushima-u.ac.jp (T.T.)

**Keywords:** bipyridine, phenanthroline, naphthyridine, ruthenium, carbonyl complex, isomerization, crystal structure

## Abstract

The reactivities of transition metal coordination compounds are often controlled by the environment around the coordination sphere. For ruthenium(II) complexes, differences in polypyridyl supporting ligands affect some types of reactivity despite identical coordination geometries. To evaluate the synergistic effects of (i) the supporting ligands, and (ii) the coordination geometry, a series of dicarbonyl–ruthenium(II) complexes that contain both asymmetric and symmetric bidentate polypyridyl ligands were synthesized. Molecular structures of the complexes were determined by X-ray crystallography to distinguish their steric configuration. Structural, computational, and electrochemical analysis revealed some differences between the isomers. Photo- and thermal reactions indicated that the reactivities of the complexes were significantly affected by both their structures and the ligands involved.

## 1. Introduction

Polypyridines with multiple covalently bonded pyridine groups exhibit unique photophysical and redox properties [1,2]. Among the polypyridines, bipyridine analogues play an important role in the formation of various transition metal complexes as bidentate ligands with two nitrogen donor atoms [3]. Bipyridine analogues function not only as supporting ligands for stabilizing metal complexes, but also as electron pool sites. For example, in addition to catalysts for the production of useful resources, such as in multi-electron reductions of carbon dioxide and water–gas shift reactions [4,5,6,7,8,9,10,11], bipyridine analogues are also utilized as photosensitizers [12] and phosphorescence materials [13]. A variety of studies that imparted selectivity to various reactions by strictly controlling the coordination sphere have been reported for many ruthenium complexes. Various molecular structures can be readily designed for a ruthenium center because ruthenium can take various oxidation states and can easily interact with the ligand. These structures include examples where differences in the coordination geometry dramatically changed both the electrical and photochemical properties of the complex, and therefore its catalysis for chemical reactions [14,15,16]. Many examples of the relationship between the supporting bipyridine ligands and the reactivity of metal complexes are known. For example, the relationship between the number of heteroatoms involved in the supporting ligand and the reactivity of the complex has been reported in a ruthenium complex containing bipyridine analogues [17].

**Chart 1:**
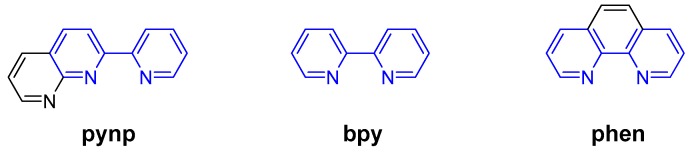
Chemical structures of pynp, bpy, and phen.

**Chart 2:**
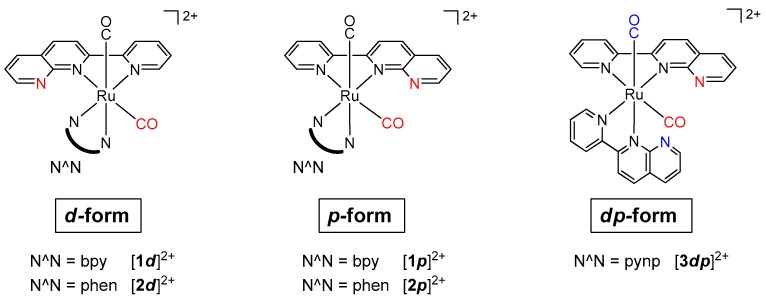
[Ru(pynp)(N^N)(CO)_2_]^2+^ complexes in this study [19].

Recently, we reported the synthesis of ruthenium complexes with 2,2’-bipyridine (bpy) and an analogue, the asymmetric bidentate ligand 2-(2-pyridyl)-1,8-naphthyridine (pynp, Chart 1). The supporting ligands affected the properties of the complexes, and diastereomers with these ligands showed different reactivities [18]. As is the case in some of the studies mentioned above, the difference in reactivities based on coordination geometry or the effect of the supporting ligand on the properties of the complex have been reported independently, but there are very few examples that investigated the synergistic effects of both. In this study, we prepared a series of Ru(II) complexes (Chart 2) containing the asymmetric bidentate pynp and a symmetric bidentate ligand N^N, [Ru(pynp)(N^N)(CO)_2_]^2+^ (N^N = bpy or 1,10-phenanthroline (phen) as a more rigid ligand, Chart 1), as a platform for investigating these synergistic effects [19]. In addition to characterizing the above complexes, we performed photo- and thermal reactions on the complexes, along with the bis–pynp complex [Ru(pynp)_2_(CO)_2_]^2+^ as a reference compound. We comprehensively evaluated the synergistic effects of the coordination geometry and supporting ligands on the reactivity.

## 2. Results and Discussion

### 2.1. Diastereoselective Synthesis and Characterization of Ruthenium Complexes with the Asymmetric pynp Ligand

#### 2.1.1. Synthesis and Structure of Complexes

**Figure 1:**
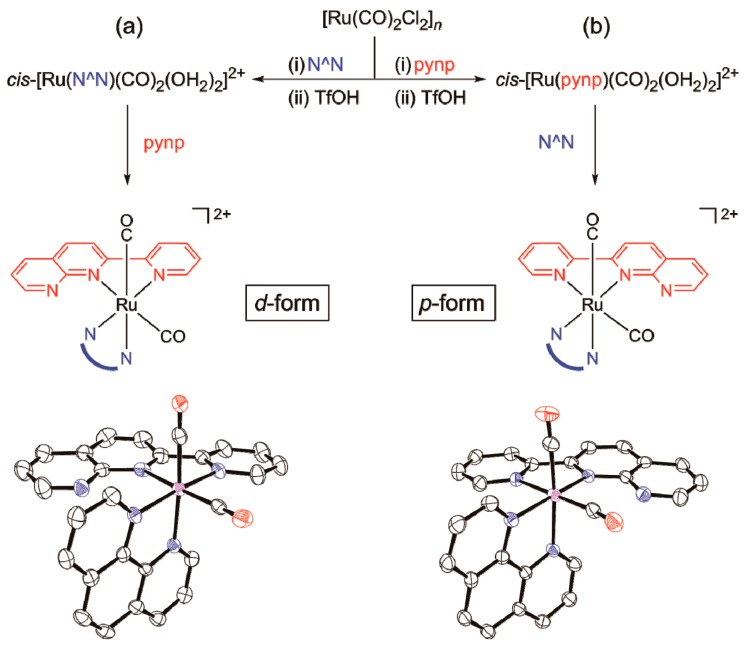
Diastereoselective synthesis of [1]^2+^ and [2]^2+^. (a) synthetic route for the *d*-isomers and molecular structure of [**2*d***]^2+^; (b) synthetic route for the *p*-isomers and molecular structure of [**2*p***]^2+^. Counteranions, H atoms, and solvent molecules are omitted for clarity. The asymmetric unit of the [**2*d***] crystal contains two chemically identical complex pairs, of which only one is displayed.

Unlike unsubstituted 2,2’-bipyridine and 1,10-phenanthroline, the pynp ligand has no *C*2 symmetry axis (Chart 1), so metal complexes containing pynp(s) may have several diastereomers. We previously reported that for ruthenium carbonyl complexes containing both bpy and pynp, formation of the desired isomers can be controlled by the order of introduction of the two ligands [18,20]. Using this method, dicarbonylruthenium complexes containing both pynp and phen were successfully prepared. As in the previous study, the distal isomer is produced preferentially when phen is introduced first (Figure 1a). In the case of N^N = bpy, the product obtained by this method is almost all the *d*-form, whereas in the phen system a small quantity of the corresponding *p*-form (10–20%) is also formed. This may be due to energy differences between the *d*- and *p*-isomers. Density functional theory (DFT) calculations for each complex system suggest that the *p*-isomers are more stable, but the energy difference between the *d*- and *p*-isomers in the phen system is smaller than that of the corresponding bpy system (N^N = bpy: 4.3 kcal/mol, N^N = phen: 1.9 kcal/mol). The DFT calculations indicate that the minor formation of the *p*-isomer in addition to the *d*-isomer is due to the relatively small energy difference in the phen system.

**Chart 3:**
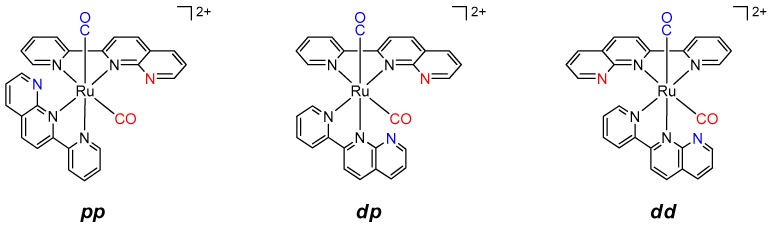
Possible diastereomers in [Ru(pynp)_2_(CO)_2_]^2+^.

**Figure 2:**
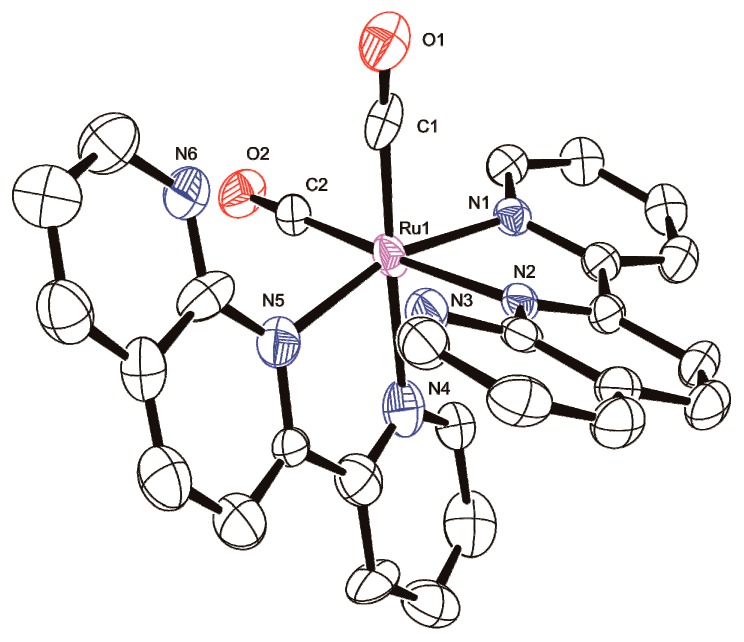
Molecular structure of *dp*-[Ru(pynp)_2_(CO)_2_]^2+^ ([**3*dp***]^2+^) with atom labels and displacement ellipsoids for non-H atoms drawn at the 50% probability level. Counteranions and H atoms are omitted for clarity.

In contrast, initial introduction of the asymmetric pynp ligand leads to the selective formation of *p*-isomers (Figure 1b). Considering the molecular structure of an isolated and analyzed intermediate *cis*(solv)-[Ru(pynp)(CO)_2_(solv)_2_]^2+^; Supplementary Materials Figure S1), it is reasonable to propose that an intramolecular interaction between the non-coordinating nitrogen atom in the pynp ligand and the adjacent coordinated carbonyl promotes the formation of *p*-isomers [18]. In addition, only the *dp*-isomer was selectively isolated in the case of [Ru(pynp)_2_(CO)_2_]^2+^ (Figure 2), even though the bis-pynp complex could form three diastereomers (*pp*, *dp*, and *dd*; Chart 3). According to DFT calculations for the three diastereomers, the total energy of the experimentally formed *dp-*isomer was 3.79 kcal/mol higher than that of the most stable *pp*-isomer, which appears to contradict the experimental result. We then performed total energy calculations for the two possible intermediates in the coordination of the second pynp ligand to the ruthenium center by assuming that the aqua ligand in the *trans* position of the coordinated pynp is substituted earlier than the aqua ligand in the *cis* position (see the structure diagram in Supplementary Materials Figure S1). The results indicate that both intermediates have almost the same energy (Δ*E* = 0.08 kcal/mol; Figure S2). Therefore, the second pynp ligand may coordinate through a kinetically favorable pathway rather than a thermodynamic one to give the selective *dp*-isomer.

Table 1 summarizes the structural parameters of a series of [Ru(pynp)(N^N)(CO)_2_]^2+^ complexes. The data around the ruthenium center and the {Ru–CO}^2+^ moieties are within the typical range of dicarbonylruthenium(II) complexes that have polypyridines as supporting ligands [4,21,22]. In addition, the interatomic distances between the carbonyl carbon and the non-coordinating nitrogen of pynp (2.607(5) to 2.754(14) Å) in the three *p*-forms are much shorter than the sum of van der Waals radii (3.25 Å) [23]. The shortening suggests that pynp and the adjacent CO ligand interact considerably in these complexes.

#### 2.1.2. Characterization of Complexes

Spectroscopic and electrochemical analyses were performed on the synthesized complexes (Table 2). Two strong IR bands assignable to νCO were observed around 2100 and 2040 cm^−1^ in all complexes. These values were similar to those in other dicarbonyl-ruthenium(II) complexes [4,21,22], and no clear differences were observed between the isomers. Given that the complexes contain two carbonyl ligands of the highest order in the spectrochemical series, no obvious absorption was observed in the visible region (Supplementary Materials Figure S3). However, intense polypyridyl-centered π–π* intraligand transitions were observed in the UV region [24].

**Figure 3:**
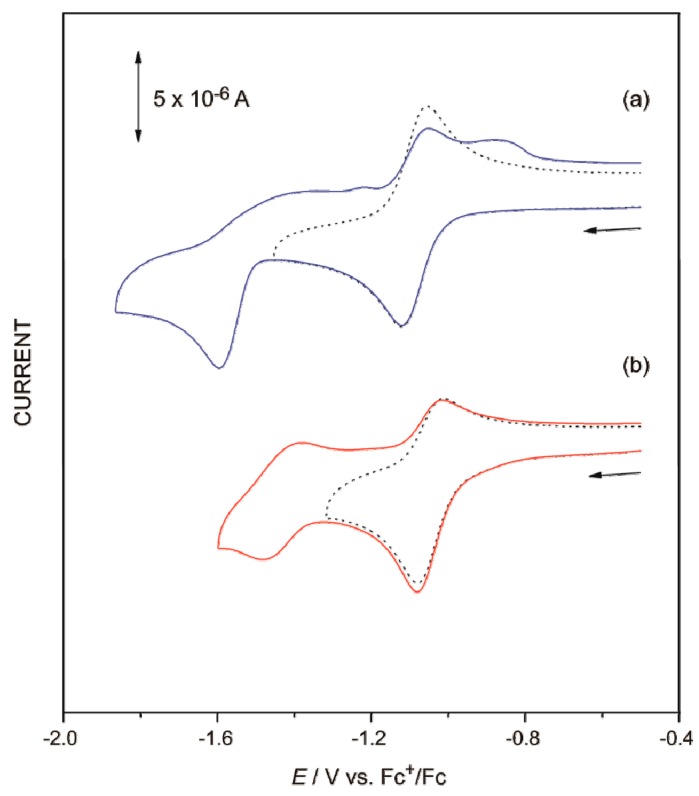
Cyclic voltammograms of (**a**) [**2*d***]^2+^ and (**b**) [**2*p***]^2+^ in CH_3_CN (*v* = 0.1 V s^−1^, *c* = 1.0 mM).

**Scheme 1:**
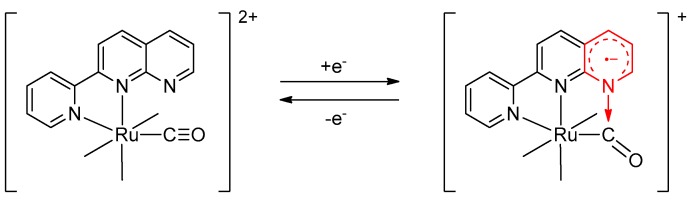
Reversible metallacyclization induced by one-electron transfer in [Ru(pynp)(CO)_2_(PPh_3_)_2_]^2+^ [26].

Although spectroscopic measurements did not show any marked differences between isomers, electrochemistry clearly showed different behavior. The cyclic voltammograms (CV) of all the complexes showed multiple ligand-based reduction waves in the range of −1 to −2 V vs. Fc^+^/Fc. Since pynp is more easily reduced than bpy or phen, the first reduction peak at ca. −1 V was attributed to the reduction of the pynp ligand [25]. When the cathodic scan was immediately reversed after the first peak potential, the coupled oxidation wave was reversible in the *d*-isomers (Figure 3a, dotted line); however, those of the corresponding *p*-isomers were either irreversible or quasi-reversible at the first reduction wave (Figure 3b). Although the first reduction wave of [**2*p***]^2+^ in Figure 3b appears to be reversible, the coupled anodic current decreased as the scan rates slowed (Supplementary Materials Figure S4). The basicity of the free nitrogen atom of the 1,8-naphthyridine moiety in pynp increased due to one-electron reduction of pynp, thus making intramolecular nucleophilic attack to the adjacent carbonyl carbons possible, leading to the formation of a metallacyclic compound (Scheme 1) [26]. Due to this structural change, only the *p*-isomer exhibited irreversible one-electron reduction behavior.

### 2.2. Reactivities of Complexes

#### 2.2.1. Photochemical Reactions

**Scheme 2:**
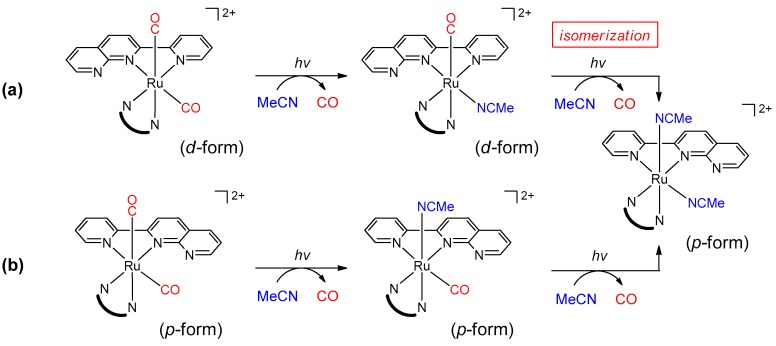
Photoreactions of [Ru(pynp)(N^N)(CO)_2_]^2+^ [29]. (**a**) The two-step reaction of *d*-isomers ([**1*d***]^2+^, [**2*d***]^2+^, and [**3*dp***]^2+^) with isomerization; (**b**) The two-step reaction of *p*-isomers ([**1*p***]^2+^ and [**2*p***]^2+^) without isomerization. The structures of these species were confirmed by X-ray crystallographic analyses (Figure 5, Supplementary Materials Figure S5, and reference [18]).

**Figure 4:**
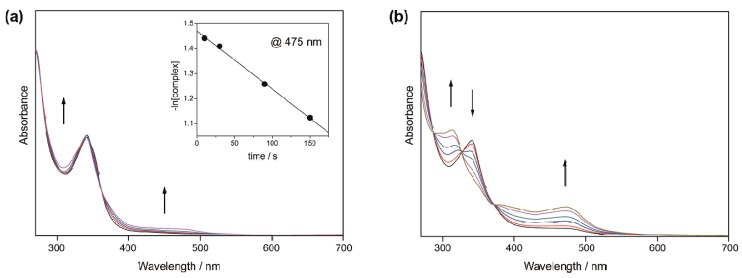
Changes in the absorption spectra of [**2*d***]^2+^ in CH_3_CN upon photoirradiation (λ = 300–400 nm). (**a**) 1st reaction step (*t* < 900 s); (**b**) 2nd reaction step (*t* > 900 s). Inset: First-order plot based on the absorbance values at 475 nm.

**Figure 5:**
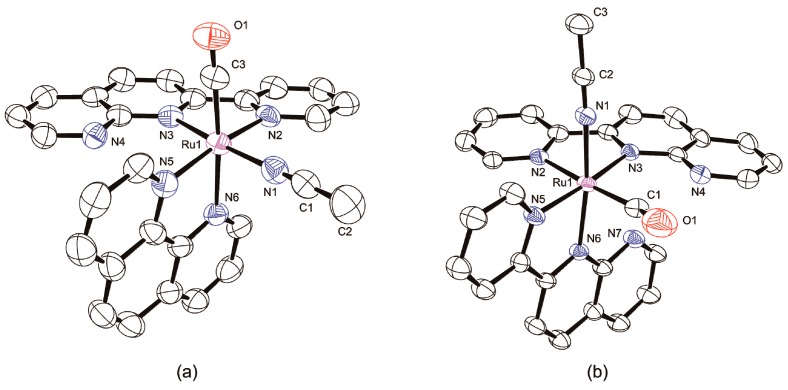
Molecular structures of monosubstituted complexes with atom labels and displacement ellipsoids for non-H atoms drawn at the 50% probability level. Counteranions, H atoms, and solvent molecules are omitted for clarity. (**a**) *d*-[Ru(pynp)(phen)(CO)(CH_3_CN)]^2+^; (**b**) *dp*-[Ru(pynp)_2_(CO)(CH_3_CN)]^2+^.

Polypyridylruthenium complexes are generally photoreactive. When *cis*-[Ru(bpy)_2_(CO)_2_]^2+^ in acetonitrile is irradiated with light, two carbonyl ligands simultaneously dissociate and the corresponding solvent complex (*cis*-[Ru(bpy)_2_(CH_3_CN)_2_]^2+^) is produced [27,28]. However, when pynp is substituted for one of the two bpy ligands, the two carbonyl groups dissociate stepwise due to their electronic non-equivalence [18]. Since no comparisons between the *d*- and *p-*isomers were made in the previous report, we compared the photoreactivities of both isomers. As previously reported, there are two reaction steps for both isomers (Scheme 2) [18,29]. In the first step, one carbonyl ligand was dissociated, and, at the same time, the acetonitrile used as the solvent became coordinated (the first step in Scheme 2 and Figure 4a). Steric retention of the complex was supported by structural analysis of the isolated species at this step (Figure 5a). In the subsequent step, photoisomerization from the *d-* to *p*-isomer subsequent slow dissociation of the second CO ligand and solvent coordination occurred (the second step in Scheme 2a and Figure 4b). Reaction analysis and DFT calculations suggest that the formation of photoexcited states of the complex promote such an isomerization to a more stable *p*-form in similar complexes containing pynp [30,31]. In the bis-pynp complex ([**3*dp***]^2+^), a similar two-step photoreaction (Scheme 2a) was confirmed from structural analyses of both products (*dp*-form in Figure 5b and *pp*-form in Supplementary Materials Figure S5). On the other hand, the final structure of the *p*-isomers was unchanged from spectroscopic analyses (Scheme 2b).

From comparisons of the first CO-dissociation rates (Table 3), it was found that the photoreactions of the *d*-isomers were always faster than those of the corresponding *p*-isomers (ca. two times in both complexes). This difference was interpreted to be due to their geometries, based on photoreaction behavior seen in similar diastereomers [32]. The pynp ligand has a naphthyridine unit in place of the pyridine unit in bpy (or phen), which is both more delocalized and a π-acceptor. The superior charge acceptor properties of the naphthyridine unit in the *d*-isomers leads to better labilization of the *trans*-CO, and thus the *d*-isomers exhibit faster CO release compared with the corresponding *p*-isomers, despite having lower extinction coefficients between 300 and 400 nm (Table 2 and Supplementary Materials Figure S3). We also found that the overall photoreaction of the bpy system proceeded more smoothly than that of the corresponding phen system. This was probably because phen compounds were significantly more stable than their bpy analogues, based on the rigidity of phen [33]. Despite prolonged photoirradiation, incomplete dissociation of the second CO ligand in the phen system consequently prevented isolation of the single disubstituted complex ([Ru(pynp)(phen)(CH_3_CN)_2_]^2+^).

#### 2.2.2. Thermochemical Reactions

**Scheme 3:**
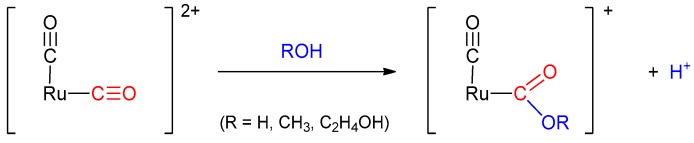
Nucleophilic attack of ROH on the coordinated CO in dicarbonyl-ruthenium(II) complexes.

**Figure 6:**
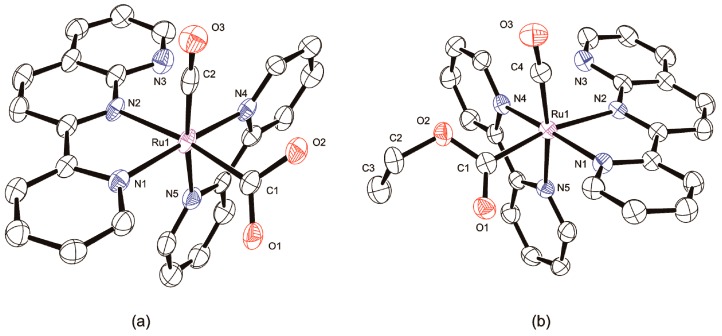
Molecular structures with atom labels and displacement ellipsoids for non-H atoms drawn at the 50% probability level. Counteranions, H atoms, and solvent molecules are omitted for clarity. (**a**) *d*-[Ru(pynp)(bpy)(CO)(C(O)OH)]^+^; (**b**) *d*-[Ru(pynp)(bpy)(CO)(C(O)OC_2_H_5_)]^+^.

As shown in Scheme 3, in thermal reactions one carbonyl ligand of a dicarbonylruthenium(II) complex undergoes nucleophilic attack from solvent molecules [34,35]. Thus, when the *d*-isomers of the dicarbonyl complexes, which are expected to be more reactive (see Section 2.2.1.), were heated in water/acetonitrile or alcohol (methanol or ethanol)/acetonitrile mixtures, one of the coordinated CO moieties underwent nucleophilic attack from the solvent to the CO ligand at the *trans* position of pynp. As expected, molecular structures of the isolated complexes showed the formation of the hydroxycarbonyl (–C(O)OH in Figure 6a) and the methoxy- or ethoxycarbonyl (–C(O)OC_2_H_5_ in Figure 6b and Supplementary Materials Figure S6) complexes. Given that the pynp ligand is more π-acidic than bpy or phen, the CO carbon in the *trans* position of pynp has a more positive charge.

**Figure 7:**
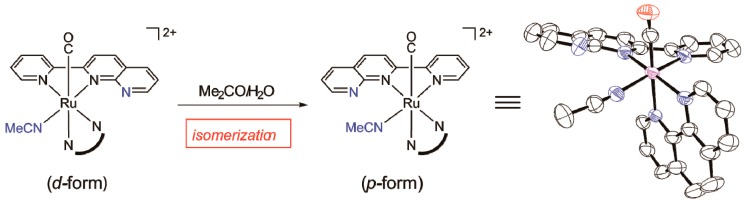
Thermal isomerization of *d*-[Ru(pynp)(N^N)(CO)(CH_3_CN)]^2+^ and the molecular structure of the isomerized *p*-form (counteranions, H atoms, and solvent molecules are omitted for clarity).

We next investigated other thermochemical reactions using monocarbonyl complexes that do not undergo nucleophilic attack on the coordinated carbonyl. When the monocarbonyl complex (*d*-[Ru(pynp)(phen)(CO)(CH_3_CN)]^2+^), which was produced by the photoreaction described in Section 2.2.1, was heated in acetone, it isomerized from the *d*- to the *p*-isomer (Figure 7). Notably, this thermal isomerization reaction was accelerated more than 70 times when water was added to the solution. A similar isomerization was observed in *d*-[Ru(pynp)(bpy)(CO)(CH_3_CN)]^2+^. In contrast, such thermal isomerization could not be confirmed for other monocarbonyl complexes with anionic ligands (–COR^−^; R^−^ = OH, OCH_3_, or OC_2_H_5_). These results strongly suggest that the thermal isomerization reaction involves a donor-acceptor interaction between the solvent and the complex. In ruthenium(II) complexes, terminal CO ligands that interact with the donor solvent tend to exhibit νCO IR frequencies over 2000 cm^−1^ [36,37]. In this study, the CO stretching frequency of the thermally isomerized acetonitrile complexes exceeded 2000 cm^−1^ (2008 and 2009 cm^−1^), while those of complexes containing an anionic ligand were 1950–1960 cm^−1^. It would therefore be expected that weak interactions between the coordinated carbonyl and the solvent (acetone or water) could induce thermal isomerization. That is, the interaction between the coordinated CO and the solvent significantly changes the electronic state of the ruthenium center, resulting in lowering the activation energy for isomerization of the *d*-isomer to the thermally more stable *p*-isomer [38].

## 3. Materials and Methods

### 3.1. General Remarks

All chemicals were purchased from commercial sources and used without further purification unless otherwise stated. All solvents used for the syntheses were anhydrous. Acetonitrile for electrochemical measurements was purified by passing through solvent purification columns (Glass Contour, Laguna, CA, USA). Then 2-(2-pyridyl)-1,8-naphthyridine (pynp), [Ru(CO)_2_Cl_2_]*_n_*, *trans*-(Cl)-[Ru(bpy)(CO)_2_Cl_2_], *trans*-(Cl)-[Ru(phen)(CO)_2_Cl_2_], *cis*(OH_2_)-[Ru(bpy)(CO)_2_(OH_2_)_2_](CF_3_SO_3_)_2_, *cis*-(OH_2_)-[Ru(phen)(CO)_2_(OH_2_)_2_](CF_3_SO_3_)_2_, and *cis*-(OH_2_)-[Ru(pynp)(CO)_2_(OH_2_)_2_](CF_3_SO_3_)_2_ were prepared according to previously reported procedures [18,39,40].

IR spectra were obtained using a JASCO FT–IR 4100 spectrometer (Tokyo, Japan). Electrospray ionization mass spectrometry (ESI–MS) data were obtained using a Bruker Daltonics micrOTOF spectrometer. Electronic spectra were obtained on a JASCO V-560 spectrophotometer. The ^1^H and ^13^C{^1^H}-NMR spectra were acquired on a JEOL JMN-AL300 spectrometer (Tokyo, Japan) operating at ^1^H and ^13^C frequencies of 300 and 75.5 MHz, respectively. Elemental analysis data were obtained on a PerkinElmer 2400II series CHN analyzer (Yokohama, Japan). Electrochemical measurements were performed on an electrochemical analyzer (ALS/CHI model 660E, Tokyo, Japan) with a solution of the complex in acetonitrile (1 mM) and *n*-Bu_4_NClO_4_ (0.1 M) as a supporting electrolyte in a cell consisting of a glassy carbon working electrode (*ϕ* = 1.6 mm), a Pt wire counter electrode, and Ag/AgNO_3_ (0.01 M) as the reference electrode. All potentials are reported in volts versus the ferrocenium/ferrocene couple (Fc^+^/Fc) under Ar at 25 °C. DFT calculations were performed using the quantum computation software *Gaussian 09* [41]. The geometries of the Ru complexes were fully optimized using a restricted DFT method employing the B3LYP function [42,43], with a 6-31G(d) basis set for the light elements (H, C, N, and O) [44,45] and a LanL2DZ basis set [46] for the Ru atom. The solvent effect of acetonitrile was evaluated using an implicit solvent model and a polarizable continuum model. The vibrational analyses were performed at the same calculation level employed for geometry optimization.

### 3.2. Synthesis of Complexes

#### 3.2.1. Synthesis of the Distal Isomers ([1d](PF_6_)_2_ and [2d](PF_6_)_2_)

[Ru(bpy)(CO)_2_(OH_2_)_2_](CF_3_SO_3_)_2_ (53 mg, 0.082 mmol) and pynp (20 mg, 0.098 mmol) were added to methanol (10 mL). The mixture was refluxed with stirring for 1.5 h. The reaction vessel was cooled to room temperature. The reaction mixture was condensed to 3 mL under reduced pressure. A light orange precipitate formed on addition of a saturated aqueous solution of KPF_6_ to the mixture. The product was collected by filtration, washed with cold water and diethyl ether, and dried in vacuo to obtain the product (56 mg, 84%). Anal. Calcd for [**1*d***](PF_6_)_2_: C_25_H_17_N_5_O_2_F_12_P_2_Ru: C, 37.05; H, 2.11; N, 8.64. Found: C, 37.40; H, 2.03; N, 8.51. ESI–MS (CH_3_CN): *m/z* 260.5 ([M]^2+^), 246.5 ([M–CO]^2+^). IR (KBr): 2097, 2044 cm^−1^ (νCO). ^1^H-NMR (acetone-*d*_6_): δ 9.76–9.71 (m, 2H), 9.25 (d, *J* = 8.4 Hz, 1H), 9.09–8.99 (m, 2H), 8.77–8.71 (m, 2H), 8.64 (dd, *J* = 6.6, 1.8 Hz, 2H), 8.57 (td, *J* = 6.3, 1.5 Hz, 1H), 8.49 (dd, *J* = 1.8, 1.5 Hz, 1H), 8.28–8.25 (m, 2H), 8.17–8.13 (m, 1H), 7.76–7.69 (m, 2H), 7.66–7.63 (m, 1H). ^13^C{^1^H}-NMR (acetone-*d*_6_): δ 192.08 and 191.49 (CO), 158.89, 158.73, 157.02, 156.71, 156.61, 156.16, 150.19, 145.33, 143.16, 142.76, 141.93, 139.61, 131.27, 130.70, 129.41, 129.07, 128.95, 127.20, 126.18, 125.92, 125.49, 125.39, 122.23. A similar reaction between [Ru(phen)(CO)_2_(OH_2_)_2_](CF_3_SO_3_)_2_ (60 mg, 0.089 mmol) and pynp (23 mg, 0.109 mmol) gave a mixture of both [**2*d***](PF_6_)_2_ and [**2*p***](PF_6_)_2_. Yield: 63 mg (84%). The crude product was recrystallized from a mixture of acetonitrile, acetone, and diethyl ether. Anal. Calcd for [**2*d***](PF_6_)_2_: C_27_H_17_N_5_O_2_F_12_P_2_Ru·CH_3_CN·0.5C_2_H_6_CO: C, 40.50; H, 2.56; N, 9.29. Found: C, 40.38; H, 2.29; N, 9.14. ESI–MS (CH_3_CN): *m/z* 272.6 ([M]^2+^), 258.6 ([M–CO]^2+^). IR (KBr): 2099, 2045 cm^−1^ (νCO). ^1^H-NMR (acetone-*d*_6_): δ 10.16 (dd, *J* = 5.1, 1.2 Hz, 1H), 9.80 (dd, *J* = 5.8, 0.9 Hz, 1H), 9.28 (dd, *J* = 7.6, 0.9 Hz, 1H), 9.18 (dd, *J* = 8.5, 1.2 Hz, 1H), 8.99 (s, 2H), 8.87 (dd, *J* = 8.3, 1.2 Hz, 1H), 8.80 (td, *J* = 8.1, 1.5 Hz, 1H), 8.52–8.47 (m, 2H), 8.39–8.23 (m, 4H), 8.08 (dd, *J* = 5.4, 1.5 Hz, 1H), 7.95 (dd, *J* = 8.1, 5.4 Hz, 1H), 7.60 (dd, *J* = 8.4, 4.5 Hz, 1H). ^13^C{^1^H}-NMR (acetone-*d*_6_): δ 192.33 and 191.47 (CO), 160.51, 159.49, 158.91, 157.29, 156.00, 155.95, 151.31, 147.34, 147.26, 145.17, 143.21, 141.68, 140.94, 139.41, 132.07, 132.06, 131.24, 129.16, 129.10, 128.46, 127.63, 127.59, 125.94, 125.83, 122.27.

#### 3.2.2. Synthesis of the Proximal Isomers ([1p](PF_6_)_2_ and [2p](PF_6_)_2_)

Using a protocol similar to that described for the synthesis of [**1*d***](PF_6_)_2_, the reaction between [Ru(pynp)(CO)_2_(OH_2_)_2_](CF_3_SO_3_)_2_ (106 mg, 0.161 mmol) and bpy (31 mg, 0.197 mmol) or phen (39 mg, 0.197 mmol) gave [**1*p***](PF_6_)_2_ or [**2*p***](PF_6_)_2_ in 81% and 86% yield, respectively. Anal. Calcd for [**1*p***](PF_6_)_2_: C_25_H_17_N_5_O_2_F_12_P_2_Ru: C, 37.05; H, 2.11; N, 8.64. Found: C, 37.22; H, 2.01; N, 8.60. ESI–MS (CH_3_CN): *m/z* 260.5 ([M]^2+^), 246.5 ([M–CO]^2+^). IR (KBr): 2097, 2045 cm^−1^ (νCO). ^1^H-NMR (acetone-*d*_6_): δ 9.64 (d, *J* = 5.1 Hz, 1H), 9.49 (d, *J* = 3.9 Hz, 1H), 9.36 (d, *J* = 8.4 Hz, 1H), 9.17–9.11 (m, 2H), 9.02–8.99 (m, 2H), 8.86 (d, *J* = 8.1 Hz, 1H), 8.69 (t, *J* = 3.8 Hz, 1H), 8.50 (t, *J* = 4.7 Hz, 1H), 8.35 (t, *J* = 4.8 Hz, 1H), 8.22–8.16 (m, 2H), 8.02 (d, *J* = 6.3 Hz, 1H), 7.84–7.77 (m, 2H), 7.50 (t, *J* = 7.4 Hz, 1H). ^13^C{^1^H}-NMR (acetone-*d*_6_): δ 193.12 and 192.20 (CO), 161.36, 158.14, 157.00, 156.88, 156.71, 155.91, 153.77, 151.45, 151.15, 145.42, 143.02, 142.94, 142.87, 140.57, 130.70, 130.65, 129.66, 128.19, 127.20, 126.92, 126.76, 126.03, 122.99. Anal. Calcd for [**2*p***](PF_6_)_2_: C_27_H_17_N_5_O_2_F_12_P_2_Ru·0.5CH_3_CN·0.5C_2_H_6_CO: C, 40.08; H, 2.45; N, 8.72. Found: C, 39.90; H, 2.17; N, 8.68. ESI–MS (CH_3_CN): *m/z* 272.6 ([M]^2+^), 258.6 ([M–CO]^2+^). IR (KBr): 2093, 2044 cm^−1^ (νCO). ^1^H-NMR (acetone-*d*_6_): δ 10.04 (dd, *J* = 5.1, 1.2 Hz, 1H), 9.48 (dd, *J* = 3.9, 1.8 Hz, 1H), 9.40 (d, *J* = 8.4 Hz, 1H), 9.30 (dd, *J* = 8.4, 1.2 Hz, 1H), 9.18 (d, *J* = 8.7 Hz, 1H), 9.10 (d, *J* = 7.8 Hz, 1H), 9.03 (dd, *J* = 7.8, 1.8 Hz, 1H), 8.96 (dd, *J* = 8.4, 0.9 Hz, 1H), 8.56–8.38 (m, 4H), 8.23–8.17 (m, 2H), 7.88 (d, *J* = 4.8 Hz, 1H), 7.82 (dd, *J* = 9.0, 5.4 Hz, 1H), 7.60 (dd, *J* = 7.5, 1.2 Hz, 1H). ^13^C{^1^H}-NMR (acetone-*d*_6_): δ 192.08 (CO), 161.61, 158.85, 157.05, 156.69, 153.96, 152.34, 151.63, 147.09, 146.35, 145.47, 142.88, 142.05, 141.81, 140.59, 132.99, 132.47, 130.39, 129.46, 129.38, 128.88, 128.13, 127.84, 127.19, 126.77, 123.01.

#### 3.2.3. Synthesis of [3dp](PF_6_)_2_

A similar reaction between [Ru(pynp)(CO)_2_(OH_2_)_2_](CF_3_SO_3_)_2_ (58 mg, 0.083 mmol) and pynp (20 mg, 0.098 mmol) in methanol (20 mL) gave [**3*dp***](PF_6_)_2_. Yield: 67 mg (94%). Anal. Calcd for [**3*dp***](PF_6_)_2_: C_28_H_18_N_6_O_2_F_12_P_2_Ru·H_2_O: C, 38.23; H, 2.29; N, 9.56. Found: C, 37.95; H, 1.94; N, 9.32. ESI–MS (CH_3_CN): *m/z* 286.1 ([M]^2+^), 272.1 ([M–CO]^2+^). IR (KBr): 2094, 2040 cm^−1^ (νCO). ^1^H-NMR (acetone-*d*_6_): δ 9.85 (d, *J* = 4.8 Hz, 1H), 9.49 (dd, *J* = 2.2, 1.8 Hz, 1H), 9.28 (d, *J* = 8.4 Hz, 2H), 9.06–9.01 (m, 3H), 8.85–8.82 (m, 2H), 8.78 (t, *J* = 1.5 Hz, 1H), 8.53 (dd, *J* = 6.6, 1.8 Hz, 1H), 8.39–8.16 (m, 3H), 7.88 (d, *J* = 4.8 Hz, 1H), 7.75 (t, *J* = 1.5 Hz, 1H), 7.66 (dd, *J* = 2.2, 1.8 Hz, 1H), 7.54–7.49 (m, 1H). ^13^C{^1^H}-NMR (acetone-*d*_6_): δ 192.58 and 192.27 (CO), 160.76, 160.25, 158.70, 157.76, 157.19, 155.92, 155.86, 151.01, 145.17, 144.22, 142.97, 142.70, 140.27, 139.57, 139.51, 131.42, 130.27, 129.16, 127.64, 127.58, 126.57, 126.11, 125.68, 125.63, 122.23, 122.03.

### 3.3. Photochemical Reactions

Photochemical reactions of the complexes were conducted in the degassed CH_3_CN. The solution was irradiated with UV–visible light (λ = 300–400 nm) through a cutoff filter using an Asahi Spectra MAX-302 (Tokyo, Japan) with a xenon lamp (0.1 mW cm^−2^). In a typical reaction, an acetonitrile solution of [**1*d***](PF_6_)_2_ (0.10 mM) was placed in a quartz flask with a stopper and irradiated with a xenon lamp for 30 min. The reaction mixture was condensed to 1 mL under reduced pressure. Addition of diethyl ether to the solution resulted in the formation of a precipitate of the mono- or disubstituted complexes in moderate yields (65–75%). 

*d*-[Ru(pynp)(bpy)(CO)(CH_3_CN)](PF_6_)_2_: ESI–MS (CH_3_CN): *m/z* 246.5 ([M–CH_3_CN]^2+^). IR (KBr): 2009 cm^−1^ (νCO). Electronic spectrum (CH_3_CN): λ_max_/nm (ε/M^−1^ cm^−1^) 250 (31,200), 312 (18,500), 341 (19,400), 480 (950). *p*-[Ru(pynp)(bpy)(CH_3_CN)_2_](PF_6_)_2_: ESI–MS (CH_3_CN): *m/z* 232.5 ([M–(CH_3_CN)_2_]^2+^). Electronic spectrum (CH_3_CN): λ_max_/nm (ε/M^−1^ cm^−1^) 239 (26,400), 288 (33,600), 407 (4400), 473 (6500). *d*-[Ru(pynp)(phen)(CO)(CH_3_CN)](PF_6_)_2_: ESI–MS (CH_3_CN): *m/z* 258.5 ([M–CH_3_CN]^2+^). IR (KBr): 2008 cm^−1^ (νCO). Electronic spectrum (CH_3_CN): λ_max_/nm (ε/M^−1^ cm^−1^) 268 (33,400), 341 (16,700), 440 (1600). *dp*-[Ru(pynp)_2_(CO)(CH_3_CN)](PF_6_)_2_: ESI–MS (CH_3_CN): *m/z* 272.0 ([M–CH_3_CN]^2+^), 292.5 ([M]^2+^). IR (KBr): 2007 cm^−1^ (νCO). Electronic spectrum (CH_3_CN): λ_max_/nm (ε/M^−1^ cm^−1^) 241 (40,200), 340 (32,000), 420_sh_ (2600). *pp*-[Ru(pynp)_2_(CH_3_CN)_2_](PF_6_)_2_: ESI–MS (CH_3_CN): *m/z* 258.0 ([M–(CH_3_CN)_2_]^2+^), 278.5 ([M–CH_3_CN]^2+^). Electronic spectrum (CH_3_CN): λ_max_/nm (ε/M^−1^ cm^−1^) 315 (40,900), 496 (6400). Kinetic measurements for the photoreaction were followed at an appropriate wavelength for each complex (476 nm for [**1*d***]^2+^, 478 nm for [**1*p***]^2+^, 475 nm for [**2*d***]^2+^ and [**2*p***]^2+^, and 497 nm for [**3*dp***]^2+^), and the logarithm of the complex concentration versus time plots were generated for the first step of the reaction in each measurement.

### 3.4. Thermochemical Reactions

#### 3.4.1. Reaction of the Coordinated CO in [1d]^2+^ and [2d]^2+^ with Solvents

The [**1*d***](PF_6_)_2_ (50 mg, 0.0617 mmol) was dissolved in acetonitrile (5 mL) and a suitable cosolvent (water, ethanol, or methanol) (30 mL) and refluxed for 24 h. The volume was reduced to 1 mL using a rotary evaporator. Addition of diethyl ether to the solution resulted in precipitation of the reaction products ([Ru(pynp)(bpy)(CO)(C(O)R)]PF_6_, R = OH, OC_2_H_5_, or OCH_3_) in moderate to high yields (50%–82%). [Ru(pynp)(bpy)(CO)(C(O)OH)]PF_6_: ESI–MS (CH_3_CN): *m/z* 538.1 ([M]^+^). IR (KBr): 1957 cm^−1^ (νCO). [Ru(pynp)(bpy)(CO)(C(O)OC_2_H_5_)]PF_6_: ESI–MS (CH_3_CN): *m/z* 566.1 ([M]^+^). IR (KBr): 1954 cm^−1^ (νCO). [Ru(pynp)(bpy)(CO)(C(O)OCH_3_)]PF_6_: ESI–MS (CH_3_CN): *m/z* 552.1 ([M]^+^). IR (KBr): 1954 cm^−1^ (νCO).

[Ru(pynp)(phen)(CO)(C(O)R)](PF_6_) complexes were also isolated in 50 to 75% yields using a similar procedure starting from [**2*d***](PF_6_)_2_. [Ru(pynp)(phen)(CO)(C(O)OH)]PF_6_: ESI–MS (CH_3_CN): *m/z* 562.0 ([M]^+^). IR (KBr): 1960 cm^−1^ (νCO). [Ru(pynp)(phen)(CO)(C(O)OC_2_H_5_)]PF_6_: ESI–MS (CH_3_CN): *m/z* 590.2 ([M]^+^). IR (KBr): 1961 cm^−1^ (νCO). [Ru(pynp)(phen)(CO)(C(O)OCH_3_)]PF_6_: ESI–MS (CH_3_CN): *m/z* 576.1 ([M]^+^). IR (KBr): 1960 cm^−1^ (νCO).

#### 3.4.2. Thermal-induced Isomerization Using Monocarbonyl Complexes *d*-[Ru(pynp)(N^N)(CO)(CH_3_CN)]^2+^ (N^N = bpy or phen) and *dp*-[Ru(pynp)_2_(CO)(CH_3_CN)]^2+^

In a typical reaction, *d*-[Ru(pynp)(phen)(CO)(CH_3_CN)](PF_6_)_2_ (4 mg, 0.005 mmol) was dissolved in acetone (20 mL) and refluxed until the solution became dark red (~72 h). The solution was condensed using a rotary evaporator, and addition of diethyl ether resulted in the formation of a precipitate. After cooling overnight, the isomerized product was collected by filtration, washed with diethyl ether, and then dried in vacuo. The yield was 3 mg (75%). A change of the solvent (acetone/water, 1:20 *v/v*) brought about a considerable reduction in reaction time (1 h). Similar reactions using *d*-[Ru(pynp)(bpy)(CO)(CH_3_CN)](PF_6_)_2_ or *dp*-[Ru(pynp)_2_(CO)(CH_3_CN)](PF_6_)_2_ gave the corresponding *p*- or *pp*-isomer, respectively. These products were characterized by spectroscopic and X-ray crystallographic analyses. 

*p*-[Ru(pynp)(bpy)(CO)(CH_3_CN)](PF_6_)_2_: ESI–MS (CH_3_CN): *m/z* 246.5 ([M–CH_3_CN]^2+^). IR (KBr): 1992 cm^−1^ (νCO). Electronic spectrum (CH_3_CN): λ_max_/nm (ε/M^−1^ cm^−1^) 251 (31,300), 312 (19,600), 343 (21,400), 383 (4700), 490 (1200). *p*-[Ru(pynp)(phen)(CO)(CH_3_CN)](PF_6_)_2_: ESI–MS (CH_3_CN): *m/z* 258.5 ([M–CH_3_CN]^2+^). IR (KBr): 1994 cm^−1^ (νCO). Electronic spectrum (CH_3_CN): λ_max_/nm (ε/M^−1^ cm^−1^) 268 (34,500), 332_sh_ (11,000), 521 (2200). *pp*-[Ru(pynp)_2_(CO)(CH_3_CN)](PF_6_)_2_: ESI–MS (CH_3_CN): *m/z* 272.0 ([M–(CH_3_CN)]^2+^), 292.5 ([M]^2+^). IR (KBr): 1994 cm^−1^ (νCO). Electronic spectrum (CH_3_CN): λ_max_/nm (ε/M^−1^ cm^−1^) 245 (31,200), 269 (26,900), 343 (28,800), 423_sh_ (3000), 499 (1000).

### 3.5. X-ray Crystallographic Analyses

Single crystals of [**2*d***]_2_(PF_6_)_3_(CF_3_SO_3_)·H_2_O, [**2*p***](PF_6_)_2_·CH_3_CN, [**3*dp***](CF_3_SO_3_)_2_, and *d*-[Ru(pynp)(phen)(CO)(CH_3_CN)](PF_6_)_2_ were obtained by vapor diffusion of diethyl ether into an acetonitrile/acetone solution of the complex. *p*-[Ru(pynp)(phen)(CO)(CH_3_CN)](PF_6_)_2_·CH_3_OH crystals were obtained by vapor diffusion of diethyl ether into an acetonitrile/acetone/methanol solution of the complex. [Ru(pynp)(CO)_2_(OH_2_)(CH_3_CN)](CF_3_SO_3_)_2_·C_2_H_5_OH crystals were obtained by vapor diffusion of diethyl ether into an acetonitrile/acetone/ethanol solution of the complex. Single crystals of *dp*-[Ru(pynp)_2_(CO)(CH_3_CN)](PF_6_)_2_·(CH_3_)_2_CO and [Ru(pynp)(bpy)(CO)(C(O)OH)]PF_6_·(CH_3_)_2_CO were obtained by vapor diffusion of diethyl ether into an acetone solution of the complex. Single crystals of *pp*-[Ru(pynp)_2_(CH_3_CN)_2_](PF_6_)_2_ were obtained by vapor diffusion of diethyl ether into an acetonitrile solution of the complex. Single crystals of *d*-[Ru(pynp)(bpy)(CO)(C(O)OC_2_H_5_)]PF_6_·(CH_3_)_2_CO and *d*-[Ru(pynp)(phen)(CO)(C(O)OC_2_H_5_)]CF_3_SO_3_ were obtained by layering diethyl ether over an acetone/ethanol solution of the complex. Crystal structure determination and refinement data for the complexes are given in Figure 1, Figure 2, Figure 5, Figure 6 and Figure 7, Supplementary Materials Figures S1, S5 and S6, and Table 4, Table 5 and Table 6 and Supplementary Materials Table S1. CCDC-1966877–1966887 contains the supplementary crystallographic data for this paper. 

## 4. Conclusions

This study successfully formed non-equivalent CO-ligand environments by lowering the molecular symmetry of the well-known benchmark complex, [Ru(bpy)_2_(CO)_2_]^2+^. In addition to the diastereoselective synthesis of the desired complexes, the relationship between reaction selectivity and coordination geometry was demonstrated using redox properties. We found that the monoacetonitrile complexes undergo thermal isomerization in the *d*-isomers, whereas the diacetonitrile complexes undergo photoisomerization to give the corresponding *p*-form. This study will conduct further research on synthetic chemistry, stereochemistry, and structure–reactivity relationships in metal complexes.

## Figures and Tables

**Table 1:** Selected bond and interatomic distances (Å) and angles (°) for dicarbonyl complexes.

Parameter	[1*d*]^2+ 2^	[1*p*]^2+ 3^	[2*d*]^2+^	[2*p*]^2+^	[3*dp*]^2+^
Ru–C	1.905 (3)	1.925 (11)	1.903 (9)	1.917 (4)	1.919 (10)
	1.900 (3)	1.910 (10)	1.888 (9)	1.911 (4)	1.886 (8)
			1.909 (9)		
			1.895 (10)		
C–O	1.134 (4)	1.123 (13)	1.145 (11)	1.133 (5)	1.132 (10)
	1.124 (4)	1.119 (12)	1.123 (11)	1.122 (5)	1.120 (11)
			1.133 (11)		
			1.121 (11)		
Ru–C–O	176.9 (3)	177.6 (8)	177.0 (8)	176.0 (4)	175.7 (7)
	176.6 (3)	174.3 (9)	175.5 (8)	171.6 (4)	175.6 (8)
			177.4 (8)		
			177.1 (8)		
C…N ^1^	-	2.671 (14)	-	2.607 (5)	2.754 (14)

^1^ Distance between the non-coordinating nitrogen in pynp and the CO carbon atoms. ^2^ [18]. ^3^ [20].

**Table 2:** Spectral and electrochemical data for dicarbonyl complexes.

Technique	[1*d*]^2+^	[1*p*]^2+^	[2*d*]^2+^	[2*p*]^2+^	[3*dp*]^2+^
IR (νCO/cm^−1^)	2097	2097	2099	2093	2094
	2044	2045	2045	2044	2040
UV–vis (λ_max_/nm)	339 (1.78)	343 (2.90)	342 (1.64)	349 (2.05)	336 (2.93)
(ε/×10^4^ M^−1^ cm^−1^)	314 (2.22)	315 (2.63)	273 (3.19)	272 (3.79)	
CV (*E*/V vs. Fc^+^/Fc)	−1.09 ^1^	−1.07 ^1^	−1.12 ^1^	−1.09 ^1^	−1.11 ^1^
	−1.45 ^1^	−1.48 ^1^	−1.59 ^1^	−1.49 ^1^	−1.29 ^1^
	−0.98 ^2^		−1.05 ^2^	−1.03 ^2^	−1.03 ^2^
	(−0.78) ^3^		(−0.87) ^3^		(−0.79) ^3^

^1^*E*_pc_. ^2^*E*_pa_. ^3^ Data in parentheses represent oxidation waves caused by the second reduction.

**Table 3:** Rates of the first photoreaction step (298 K) of the dicarbonyl complexes.

Rate	[1*d*]^2+^	[1*p*]^2+^	[2*d*]^2+^	[2*p*]^2+^	[3*dp*]^2+^
*k*_obs_/sec^−1^	12 × 10^−3^	6.5 × 10^−3^	2.4 × 10^−3^	1.3 × 10^−3^	7.6 × 10^−3^

**Table 4:** Crystallographic data for dicarbonyl complexes.

Parameter	[2*d*]	[2*p*]	[3*dp*]
Chemical formula	C_55_H_34_F_21_N_10_O_8_P_3_Ru_2_S	C_29_H_20_F_12_N_6_O_2_P_2_Ru	C_30_H_18_F_6_N_6_O_8_RuS_2_
Formula weight	1689.02	875.51	869.69
Temperature (K)	93	93	93
Crystal system	monoclinic	triclinic	triclinic
Space group	*P*2_1_/*c*	*P*-1	*P*-1
*a* (Å)	13.2730(3)	8.57310(10)	9.68729(18)
*b* (Å)	22.5773(6)	12.7558(3)	13.0592(3)
*c* (Å)	20.2047(5)	15.8850(4)	13.6489(3)
*α* (°)	90	82.856(6)	83.1931(7)
*β* (°)	93.7571(8)	75.919(6)	81.5465(7)
*γ* (°)	90	74.843(6)	72.0749(7)
*V* (Å^3^)	6041.7(3)	1622.90(8)	1620.10(6)
*Z*	4	2	2
Calcd density (g/cm^3^)	1.857	1.792	1.783
*μ* (Mo *Kα*) (mm^–1^)	0.744	0.691	0.709
No. unique reflns	62,024	16,768	16,909
No. obsd reflns	13,818	7331	7304
Refinement method	*Full-matrix least-squares on F^2^*		
Parameters	901	470	478
*R* (*I* > 2*σ*(*I*)) ^1^	0.0941	0.0552	0.1119
*wR* (all data) ^2^	0.2536	0.1483	0.2633
*S*	1.155	1.077	1.189

^1^*R* = Σ(||*F*_o_| − |*F*_c_||)/Σ|*F*_o_|; ^2^ *wR* = {Σ*_w_*(*F*_o_^2^ − *F*_c_^2^)^2^/Σ*_w_*(*F*_o_^2^)^2^}^1/2^.

**Table 5:** Crystallographic data for [Ru(pynp)(N^N)(CO)(CH_3_CN)]^2+^ (N^N = phen or pynp).

Parameter	*d*-(phen)	*p*-(phen)	*dp*-(pynp)
Chemical formula	C_28_H_20_F_12_N_6_OP_2_Ru	C_29_H_20_F_12_N_6_O_2_P_2_Ru	C_32_H_27_F_12_N_7_O_2_P_2_Ru
Formula weight	847.50	875.51	932.61
Temperature (K)	93	93	93
Crystal system	orthorhombic	triclinic	monoclinic
Space group	*Pna*2_1_	*P*-1	*P*2_1_/*n*
*a* (Å)	13.8734(9)	11.9822(4)	10.067(4)
*b* (Å)	24.7954(15)	12.5252(3)	32.588(12)
*c* (Å)	9.0869(5)	13.2975(4)	11.329(5)
*α* (°)	90	84.1114(11)	90
*β* (°)	90	87.5838(8)	109.861(5)
*γ* (°)	90	63.326(2)	90
*V* (Å^3^)	3125.9(3)	1773.87(9)	3496(2)
*Z*	4	2	4
Calcd density (g/cm^3^)	1.801	1.639	1.772
*μ* (Mo *Kα*) (mm^−1^)	0.712	0.632	0.648
No. unique reflns	30,815	18,631	35,054
No. obsd reflns	7100	8064	7963
Refinement method	*Full-matrix least-squares on F^2^*		
Parameters	452	436	508
*R* (*I* > 2*σ*(*I*)) ^1^	0.0506	0.0832	0.0745
*wR* (all data) ^2^	0.1237	0.2503	0.1623
*S*	1.028	1.049	1.113

^1^*R* = Σ(||*F*_o_| − |*F*_c_||)/Σ|*F*_o_|; ^2^ *wR* = {Σ*_w_*(*F*_o_^2^ − *F*_c_^2^)^2^/Σ*_w_*(*F*_o_^2^)^2^}^1/2^.

**Table 6:** Crystallographic data for [Ru(pynp)(bpy)(CO)(COR)]^+^ (R = OH or OC_2_H_5_).

Parameter	R = OH	R = OC_2_H_5_
Chemical formula	C_28_H_24_F_6_N_5_O_4_PRu	C_30_H_28_F_6_N_5_O_4_PRu
Formula weight	740.56	768.62
Temperature (K)	93	93
Crystal system	triclinic	monoclinic
Space group	*P*-1	*P*2_1_/*n*
*a* (Å)	8.35369(15)	17.2474(5)
*b* (Å)	12.9208(3)	8.1493(2)
*c* (Å)	13.3343(3)	22.1009(6)
*α* (°)	88.9616(7)	90
*β* (°)	79.0700(7)	102.3249(8)
*γ* (°)	88.2204(7)	90
*V* (Å^3^)	1412.36(5)	3034.77(14)
*Z*	2	4
Calcd density (g/cm^3^)	1.741	1.682
*μ* (Mo *Kα*) (mm^−1^)	0.697	0.652
No. unique reflns	12,245	30,159
No. obsd reflns	4934	6960
Refinement method	*Full-matrix least-squares on F^2^*	
Parameters	406	463
*R* (*I* > 2*σ*(*I*)) ^1^	0.0583	0.0475
*wR* (all data) ^2^	0.1331	0.1132
*S*	1.219	1.067

^1^*R* = Σ(||*F*_o_| − |*F*_c_||)/Σ|*F*_o_|; ^2^ *wR* = {Σ*_w_*(*F*_o_^2^ − *F*_c_^2^)^2^/Σ*_w_*(*F*_o_^2^)^2^}^1/2^.

## Supplementary Materials

The following are available online at https://www.mdpi.com/1420-3049/25/1/27/s1, Figure S1: Molecular structure of [Ru(pynp)(CO)_2_(OH_2_)(CH_3_CN)]^2+^, Figure S2: Optimized structures of the monosubstituted precursor of [3]^2+^ with the electronic energy difference, Figure S3: Electronic spectra of [**1*d***]^2+^, [**1*p***]^2+^, [**2*d***]^2+^, [**2*d***]^2+^, and [**3*dp***]^2+^, Figure S4: Cyclic voltammograms of [**2*p***]^2+^ at various scan rates, Figure S5: Molecular structure of [Ru(pynp)_2_(CH_3_CN)_2_]^2+^, Figure S6: Molecular structure of [Ru(pynp)(phen)(CO)(C(O)OC_2_H_5_)]^+^, Table S1: Crystallographic data for [Ru(pynp)(CO)_2_(OH_2_)(CH_3_CN)]^2+^, [Ru(pynp)_2_(CH_3_CN)_2_]^2+^, and [Ru(pynp)(phen)(CO)(C(O)OC_2_H_5_)]^+^.

## References

[1] Smith A.P., Fraser C.L., McClevety J.A., Meyer T.J. (2004). Bipyridine ligands. Comprehensive Coordination Chemistry II.

[2] Winter A., Hoeppener S., Newkome G.M., Schubert U.S. (2011). Terpyridine-functionalized surfaces: Redox-active, switchable, and electroactive nanoarchitectures. Adv. Mater..

[3] Constable E.C., Housecroft C.E. (2017). More hydra than Janus – Non-classical coordination modes in complexes of oligopyridine ligands. Coord. Chem. Rev..

[4] Fukushima T., Ghosh D., Kobayashi K., Ohtsu H., Kitagawa S., Tanaka K. (2016). Four-electron reduction of a new ruthenium dicarbonyl complex having two NAD model ligands through decarboxylation in water. Inorg. Chem..

[5] Kobayashi K., Tanaka K. (2015). Reactivity of CO_2_ activated on transition metals and sulfur ligands. Inorg. Chem..

[6] Machan C.W., Sampson M.D., Kubiak C.P. (2015). A molecular ruthenium electrocatalyst for the reduction of carbon dioxide to CO and formate. J. Am. Chem. Soc..

[7] Kuramochi Y., Itabashi J., Fukaya K., Enomoto A., Yoshida M., Ishida H. (2015). Unexpected effect of catalyst concentration on photochemical CO_2_ reduction by *trans*(Cl)-Ru(bpy)(CO)_2_Cl_2_: new mechanistic insight into the CO/HCOO^–^ selectivity. Chem. Sci..

[8] Kuramochi Y., Fukaya K., Yoshida M., Ishida H. (2015). *trans*-(Cl)-[Ru(5,5*’*-diamide-2,2*’*-bipyridine)(CO)_2_Cl_2_]: Synthesis, structure, and photocatalytic CO_2_ reduction activity. Chem. Eur. J..

[9] Voyame P., Toghill K.E., Méndez M.A., Girault H.H. (2013). Photoreduction of CO_2_ using [Ru(bpy)_2_(CO)L]*^n^*^+^ catalysts in biphasic solution/supercritical CO_2_ systems. Inorg. Chem..

[10] Majumdar M., Sinha A., Ghatak T., Patra S.K., Sadhukhan N., Rahaman S.M.W., Bera J.K. (2010). Mapping the transformation [{Ru^II^(CO)_3_Cl_2_}_2_] to [Ru^I^_2_(CO)_4_]^2+^: Implications in binuclear water-gas shift chemistry. Chem. Eur. J..

[11] Ishida H., Tanaka K., Morimoto M., Tanaka T. (1986). Isolation of intermediates in the water gas shift reactions catalyzed by [Ru(bpy)_2_(CO)Cl]^+^ and [Ru(bpy)_2_(CO)_2_]^2+^. Organometallics.

[12] Kumar A., Kumar P., Aathira M.S., Singh D.P., Behera B., Jain S.L. (2018). A bridged ruthenium dimer (Ru-Ru) for photoreduction of CO_2_ under visible light irradiation. Ind. End. Chem..

[13] Son A., Kawasaki A., Hara D., Ito T., Tanabe K. (2014). Phosphorescent ruthenium complexes with a nitroimidazole unit that image oxygen fluctuation in tumor tissue. Chem. Eur. J..

[14] Carrington S.J., Chakraborty I., Alvarado J.R., Mascharak P.K. (2013). Differences in the CO photolability of *cis*- and *trans*-[RuCl_2_(azpy)(CO)_2_] complexes: Effect of metal-to-ligand back-bonding. Inorg. Chim. Acta..

[15] Nakamura G., Kondo M., Crisalli M., Lee S.K., Shibata A., Ford P.C., Masaoka S. (2015). Syntheses and properties of phosphine-substituted ruthenium(II) polypyridine complexes with nitrogen oxides. Dalton Trans..

[16] Boyer J.L., Polyansky D.E., Szalda D.J., Zong R., Thummel R.P., Fujita E. (2011). Effects of a proximal base on water oxidation and proton reduction catalyzed by geometric isomers of [Ru(tpy)(pynap)(OH_2_)]^2+^. Angew. Chem. Int. Ed..

[17] Ashford D.L., Glasson C.R.K., Norris M.R., Concepcion J.J., Keinan S., Brennaman M.K., Templeton J.L., Meyer T.J. (2014). Controlling ground and excited state properties through ligand changes in ruthenium polypyridyl complexes. Inorg. Chem..

[18] Oyama D., Abe R., Takase T. (2017). CO-ligand photodissociation in two Ru(II) complexes affected by different polypyridyl supporting ligands. Chem. Lett..

[B19-molecules-25-00027] 19.The *distal* and *proximal* isomers are defined by the relative positions of the monodentate ligand (L) and non-coordinating nitrogen atom of pynp: the *d*-isomer and *p*-isomer denote *cis*(L,py)- and *trans*(L,py)-, respectively (py: pyridyl group in pynp).

[20] Oyama D., Abe R., Takase T. (2018). Coordination chemistry of mononuclear ruthenium complexes bearing versatile 1,8-naphthyridine units: Utilization of specific reaction sites constructed by the secondary coordination sphere. Coord. Chem. Rev..

[21] Ooyama D., Kobayashi T., Shiren K. (2003). Tanaka, K. Regulation of electron donating ability to metal center: isolation and characterization of ruthenium carbonyl complexes with *N,N*- and/or *N,O*-donor polypyridyl ligands. J. Organomet. Chem..

[22] Tanaka H., Tzeng B.-C., Nagao H., Peng S.-M., Tanaka K. (1993). Comparative study on crystal structures of ruthenium bipyridine carbonyl complexes [Ru(bpy)_2_(CO)_2_](PF_6_)_2_, [Ru(bpy)_2_(CO)(C(O)OCH_3_)]B(C_6_H_5_)_4_·CH_3_CN, and [Ru(bpy)_2_(CO)(η^1^-CO_2_)]·3H_2_O (bpy = 2,2*’*-bipyridyl). Inorg. Chem..

[23] Bondi A. (1964). van der Waals volumes and radii. J. Phys. Chem..

[24] Zhao H.C., Fu B.-L., Schweinfurth D., Harney J.P., Sarkar B., Tsai M.-K., Rochford J. (2013). Tuning oxyquinolate non-innocence at the ruthenium polypyridyl core. Eur. J. Inorg. Chem..

[25] Campos-Fernández C.S., Ouyang X., Dunbar K.R. (2000). A homologous series of redox-active, dinuclear cations with the bridging ligand 2-(2-pyridyl)-1,8-naphthyridine. Inorg. Chem..

[26] Oyama D., Ukawa N., Hamada T., Takase T. (2015). Reversible intramolecular cyclization in ruthenium complexes induced by ligand-centered one-electron transfer on bidentate naphthyridine: An important intermediate for both metal- and organo-hydride species. Chem. Lett..

[27] Knoll J.D., Albani B.A., Turro C. (2015). New Ru(II) complexes for dual photoreactivity: Ligand exchange and ^1^O_2_ generation. Acc. Chem. Res..

[28] Liu Y., Turner D.B., Singh T.N., Angeles-Boza A.M., Chouai A., Dunbar K.R., Turro C. (2009). Ultrafast ligand exchange: detection of a pentacoordinate Ru(II) intermediate and product formation. J. Am. Chem. Soc..

[B29-molecules-25-00027] 29.Based on isolation and identification of the intermediates, we confirmed that the photoreaction proceeds through the two-step process as shown in Scheme 2.

[30] Tanaka S., Takahashi K., Hirahara M., Yagi M., Onda K. (2015). Characterization of the excited states of *distal*- and *proximal*-[Ru(tpy)(pynp)OH_2_]^2+^ in aqueous solution using time-resolved infrared spectroscopy. J. Photochem. Photobiol. A.

[31] Hirahara M., Ertem M.Z., Komi M., Yamazaki H., Cramer C.J., Yagi M. (2013). Mechanisms of photoisomerization and water-oxidation catalysis of mononuclear ruthenium(II) monoaquo complexes. Inorg. Chem..

[32] Oyama D., Yuzuriya K., Naoi R., Hamada T., Takase T. (2014). Syntheses of geometrical isomers for comparison of properties caused by steric and electronic effects in carbonylruthenium(II) complexes. Bull. Chem. Soc. Jpn..

[33] Constable E.C., Housecroft C.E. (2019). The early years of 2,2*’*-bipyridine – A ligand in its own lifetime. Molecules.

[34] Oyama D., Hamada T., Ukawa N., Mochizuki R., Takase T. (2015). Isolation and characterization of a metallacyclic compound by selective protection of a single CO ligand in a ruthenium complex. Bull. Chem. Soc. Jpn..

[35] Haukka M., Kiviaho J., Ahlgren M., Pakkanen A.T. (1995). Studies on catalytically active ruthenium carbonyl bipyridine systems. Synthesis and structural characterization of [Ru(bpy)(CO)_2_Cl_2_], [Ru(bpy)(CO)_2_Cl(C(O)OCH_3_)], [Ru(bpy)(CO)_2_Cl]_2_ and [Ru(bpy)(CO)_2_ClH] (bpy = 2,2’-bipyridine). Organometallics.

[36] Kobayashi K., Kikuchi T., Kitagawa S., Tanaka K. (2014). Selective generation of formamides through photocatalytic CO_2_ reduction catalyzed by ruthenium carbonyl compounds. Angew. Chem. Int. Ed..

[37] Oyama D., Hamada T., Takase T. (2011). Stereospecific synthesis and redox properties of ruthenium(II) carbonyl complexes bearing a redox-active 1,8-naphthyridine unit. J. Organomet. Chem..

[38] Oyama D., Kainuma S., Akatsuka K., Abe R., Takase T. (2019). Solvent mediated complete *trans*-to-*cis* isomerization of [Ru(polypyridine)(CO)_2_Cl_2_] complexes. J. Organomet. Chem..

[39] Campos-Fernández C.S., Thomson L.M., Galán-Mascarós J.R., Ouyang X., Dunbar K.R. (2002). Homologous series of redox-active, dinuclear cations [M_2_(O_2_CCH_3_)_2_(pynp)_2_]^2+^ (M = Mo, Ru, Rh) with the bridging ligand 2-(2-pyridyl)-1,8-naphthyridine (pynp). Inorg. Chem..

[40] Anderson P.A., Deacon G.B., Haarmann K.H., Keene F.R., Meyer T.J., Reitsma D.A., Skelton B.W., Strouse G.F., Thomas N.C., Treadway J.A. (1995). Designed synthesis of mononuclear tris(heteroleptic) ruthenium complexes containing bidentate polypyridyl ligands. Inorg. Chem..

[41] Frisch M.J., Trucks G.W., Schlegel H.B., Scuseria G.E., Robb M.A., Cheeseman J.R., Scalmani G., Barone V., Mennucci B., Petersson G.A. (2009). Gaussian 09W, revision D.01.

[42] Lee C., Yang W., Parr R.G. (1988). Development of the Colle-Salvetti correlation-energy formula into a functional of the electron density. Phys. Rev. B.

[43] Becke A.D. (1993). Density-functional thermochemistry. III. The role of exact exchange. J. Chem. Phys..

[44] Francl M.M., Pietro W.J., Hehre W.J., Binkley J.S., Gordon M.S., DeFrees D.J., Pople J.A. (1982). Self-consistent molecular orbital methods. XXIII. A polarization-type basis set for second-row elements. J. Chem. Phys..

[45] Hehre W.J., Ditchfield R., Pople J.A. (1972). Self-consistent molecular orbital methods. XII. Further extensions of Gaussian-type basis sets for use in molecular orbital studies of organic molecules. J. Chem. Phys..

[46] Wadt W.R., Hay P.J. (1985). *Ab initio* effective core potentials for molecular calculations. Potentials for main group elements Na to Bi. J. Chem. Phys..

