# Supplementary material for: Coordination Chemistry of Ru(II) Complexes of an Asymmetric Bipyridine Analogue: Synergistic Effects of Supporting Ligand and Coordination Geometry on Reactivities
Source: Molecules. 2019 Dec 19;25(1):27. doi: 10.3390/molecules25010027 (PMC6983075; doi:10.3390/molecules25010027)
Supplement: Supplementary file 1 [file molecules-25-00027-s001.pdf]

Supplementary Materials

Coordination Chemistry of Ru(II) Complexes of an Asymmetric Bipyridine Analogue: Synergistic Effects of Supporting Ligand and Coordination Geometry on Reactivities

Komi Akatsuka[1], Ryosuke Abe[1], Tsugiko Takase[1] and Dai Oyama[1,*]

[1] *Cluster of Science and Engineering, Fukushima University, 1 Kanayagawa, Fukushima 960-1296, Japan*

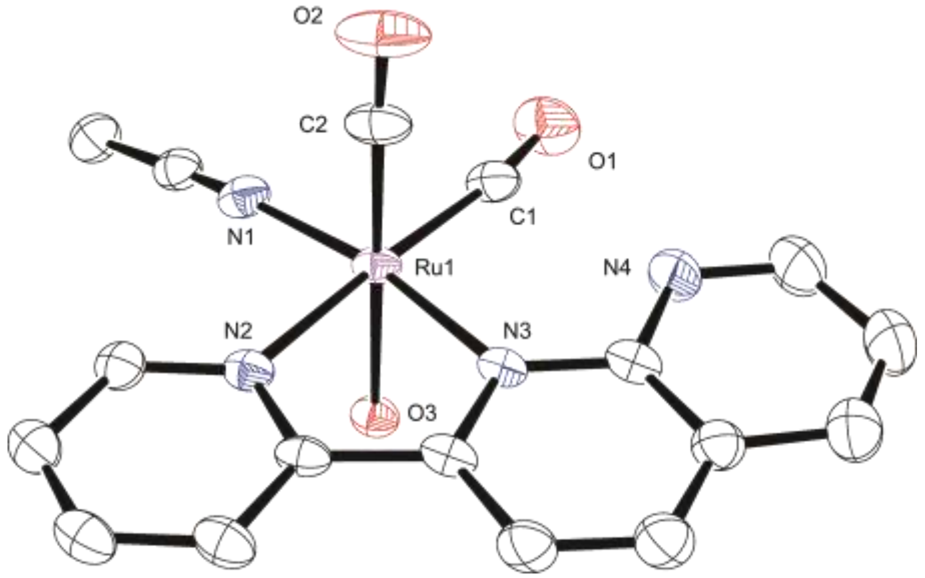

Figure S1. Molecular structure of [Ru(pynp)$(CO)_2(OH_2)(CH_3CN)]^{2+}$ (CCDC: 1966886).

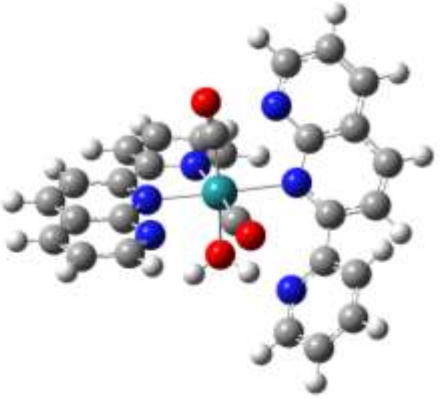

[Ru(pynp)(pynp-κN^{np})$(CO)_2(OH_2)]^{2+}$

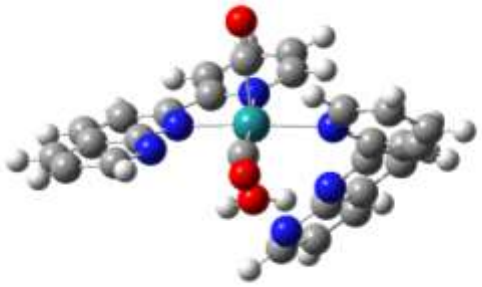

[Ru(pynp)(pynp-κN^{py})$(CO)_2(OH_2)]^{2+}$

(ΔE = +0.08 kcal/mol)

Figure S2. Optimized structures of the monosubstituted precursor of [**3**]$^{2+}$ (in CH_3CN) with the electronic energy difference.

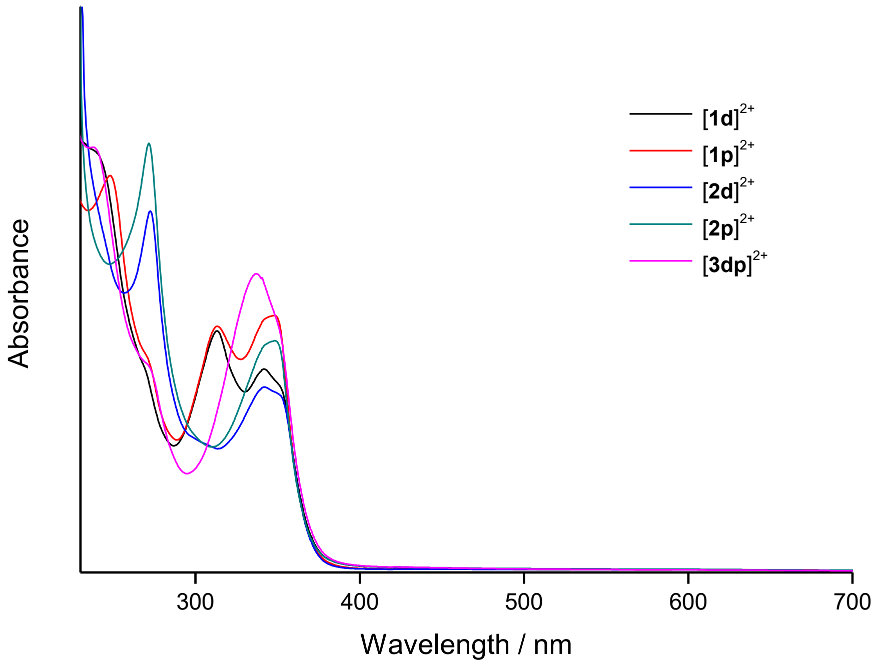

Figure S3. Electronic spectra of [**1*d***]$^{2+}$, [**1*p***]$^{2+}$, [**2*d***]$^{2+}$, [**2*d***]$^{2+}$, and [**3*dp***]$^{2+}$ (1.0×10^{-4} M in CH_3CN).

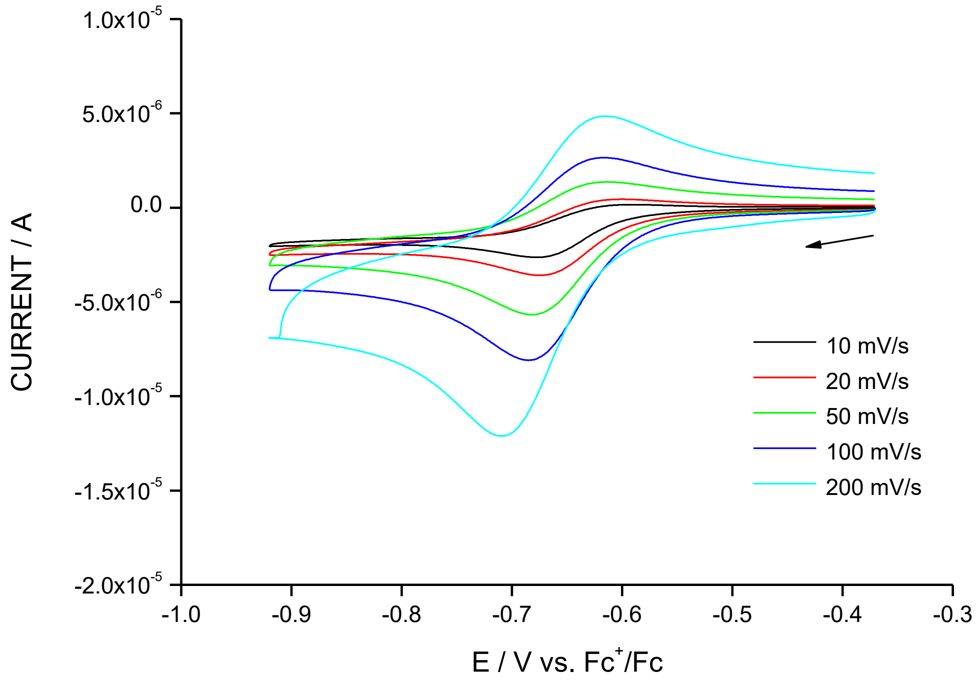

Figure S4. Cyclic voltammograms of [**2*p***]$^{2+}$ at various scan rates (10–200 mV s^{-1}).

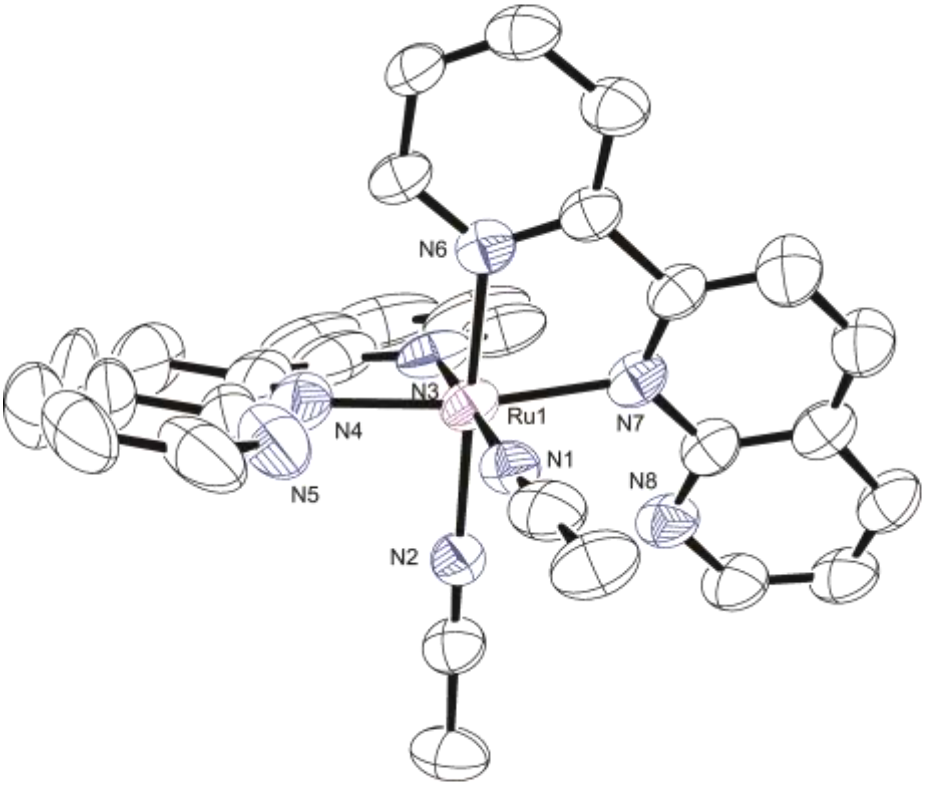

Figure S5. Molecular structure of $[Ru(pynp)_2(CH_3CN)_2]^{2+}$ (CCDC: 1966887).

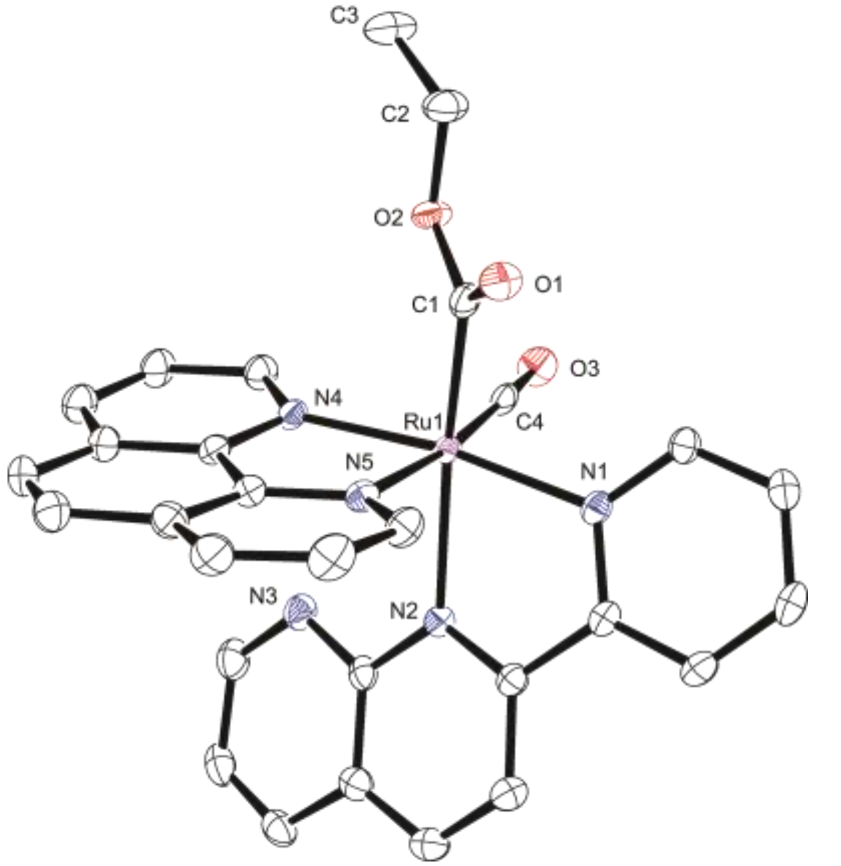

Figure S6. Molecular structure of [Ru(pynp)(phen)(CO)(C(O)OC$_2$H$_5$)]$^+$ (CCDC: 1966885).

Table S1. Crystallographic data for $[Ru(pynp)(CO)_2(OH_2)(CH_3CN)]^{2+}$ (**I**), $[Ru(pynp)_2(CH_3CN)_2]^{2+}$ (**II**), and $[Ru(pynp)(phen)(CO)(C(O)OC_2H_5)]^{+}$ (**III**).

**Parameter**	**I**	**II**	**III**
Chemical formula	$C_{21}H_{14}F_6N_4O_{10}RuS_2$	$C_{30}H_{24}F_{12}N_8P_2Ru$	$C_{30}H_{22}F_3N_5O_6RuS$
Formula weight	761.54	887.57	738.66
Temperature (K)	93	93	293
Crystal system	monoclinic	triclinic	triclinic
Space group	*C*2/*c*	*P*-1	*P*-1
a (Å)	44.0291(8)	12.044(4)	10.6283(3)
b (Å)	9.60060(17)	12.342(4)	11.5425(3)
c (Å)	13.2719(2)	13.361(4)	12.4756(3)
α (°)	90	104.282(6)	95.6201(8)
β (°)	98.5860(7)	96.558(5)	102.9741(7)
γ (°)	90	110.747(4)	104.5165(8)
V (Å^3)	5547.21(17)	1754.9(10)	1424.37(6)
Z	8	2	2
Calcd density (g/cm^3)	1.824	1.680	1.722
μ (Mo $K\alpha$) (mm^{-1})	0.817	0.638	0.699
No. unique reflns	28007	18083	15001
No. obsd reflns	6354	7904	6458
Refinement method	*Full-matrix least-squares on* F^2		
Parameters	365	534	415
R [$I > 2\sigma(I)$] [1]	0.0798	0.1055	0.0382
wR (all data) [2]	0.2131	0.2930	0.0843
S	1.064	1.025	1.078

[1] $R = \Sigma(||F_o| - |F_c||)/\Sigma|F_o|$; [2] $wR = \{\Sigma_w(F_o^2 - F_c^2)^2/\Sigma_w(F_o^2)^2\}^{1/2}$.
